# The role of social media in parents’ approaches to dental treatment procedures under general anesthesia and sedation: a cross-sectional survey in Turkey

**DOI:** 10.1186/s12903-026-07779-9

**Published:** 2026-02-02

**Authors:** Yasemin Derya Fidancıoğlu, Tuba Çatak

**Affiliations:** 1 Faculty of Dentistry, Department of Pediatric Dentistry, Necmettin Erbakan University, Beyşehir Street, Meram, Konya 42090 Turkey; 2 Faculty of Dentistry, Department of Pediatric Dentistry, MD, Anesthesiology and Reanimation, Necmettin Erbakan University, Beyşehir Street, Meram, Konya, 42090 Turkey

**Keywords:** Decision making, Dental care for children, Health literacy, Social media

## Abstract

**Background:**

The research investigates how parents use social media to decide between general anesthesia (GA) and sedation dental treatments for their children and evaluates the impact of educational level and health literacy on this decision-making process.

**Method:**

This study used a cross-sectional analytical approach to investigate 385 parents who resided in Konya province central districts. The research collected participant data about socio-demographic characteristics and social media usage habits and information-seeking behaviors through face-to-face questionnaires while conducting trustworthiness assessments. The research divided participants into subgroups based on their educational level and age and income and health literacy to evaluate treatment decision impacts and emotional changes across these groups. The researchers conducted statistical analyses through Statistical Package for the Social Sciences (SPSS) 23 software by applying Chi-square, Mann-Whitney U, Kruskal-Wallis, and Wilcoxon tests.

**Results:**

The survey revealed that 80% of participants searched for GA/sedation information on social media platforms and 64.6% of participants stated that the information they found on social media influenced their treatment choices. The use of social media platforms was associated with decreased anxiety levels from 7.18 ± 1.82 to 6.09 ± 2.28 (*p* < 0.001) while simultaneously raising confidence and satisfaction expectations (confidence: 5.31 ± 2.14 to 6.82 ± 1.94; *p* < 0.001). The trustworthiness score of university graduates (3.64 ± 0.71) exceeded that of primary school graduates (2.83 ± 0.91) at a statistically significant level (*p* < 0.001). The exposure to negative content reached 60.1% of participants and the rate of exposure grew with increasing social media usage duration (3 + hours: 66.7% vs. < 1 h: 45.0%; *p* = 0.034).

**Conclusion:**

The research demonstrates that social media was reported as a primary factor associated with parental choices. Heavy social media users experienced more negative content exposure but positive content reduced their anxiety levels. Health literacy together with education determine how people evaluate information yet the research shows that less educated individuals become more influenced by social media content.

**Supplementary Information:**

The online version contains supplementary material available at 10.1186/s12903-026-07779-9.

## Introduction

Dental treatment anxiety is a common problem in children, with 63% of children under the age of 12 experiencing moderate levels of dental anxiety and 14% experiencing severe levels [1]. This fear and anxiety pose a significant barrier to successful dental treatment and lead to serious difficulties in behaviour management. The practice of pediatric dentistry relies on pharmacological methods including conscious sedation and general anesthesia because these approaches help anxious children complete their treatments [2]. These diagnostic tools serve as vital tools for medical professionals who need to treat patients who require multiple treatments, have difficulty with behavior control, and require specialized healthcare services.

Research evidence about how parents seek information about pediatric dental anesthesia is scarce but parents commonly use online resources to find information about their children’s medical needs and treatment possibilities. The way parents locate and assess and use health information depends on their health literacy skills and their ability to recognize trustworthy sources [1–3]. The way parents understand GA/sedation practices determines their ability to give consent for treatment and their willingness to follow instructions which leads to successful clinical outcomes.

The majority of European internet users seek health-related information online because parents now rely on digital platforms to find information about their children’s health issues [3]. Social media platforms serve as essential resources which enable parents to exchange experiences with healthcare professionals while learning about their children’s oral health [4]. The internet usage rate in Turkey rose from 87.1% in 2023 to 88.8% in 2024 among people aged 16–74 while specialist care access became direct for patients without referrals thus parents must find treatment details on their own [5]. The direct-access healthcare system in Turkey requires parents to obtain treatment information independently because 66.8% of Turkish citizens use social media [6]. The city of Konya demonstrates how Turkish urban areas integrate their conventional information systems with digital resources. The majority of parents including immigrant parents choose digital platforms instead of traditional information sources which affects their health choices [7].

Research shows that parents fail to distinguish their child health information searches from their personal health inquiries while ignoring the unique characteristics of this field [3]. Parents face difficulties when evaluating online source credibility because they lack the skills to verify health information on the internet which frequently presents unreliable or deceptive content [8]. The lack of standardisation in paediatric dental health information shared on social media can lead to the spread of misinformation and cause parents to make incorrect decisions [4]. The dissemination of false information would result in patients avoiding medical care which would allow diseases to progress while resulting in adverse treatment outcomes. Furthermore, there are no clear findings on the comparative effectiveness of different sedation techniques and general anaesthesia, which complicates parents’ decision-making processes [1].

The researchers selected cross-sectional design to study present parental behaviors during the fast digital changes in healthcare communication because it enabled them to study social media usage and health literacy and decision-making at the same time. Although it has been reported that errors occur in 21% of clinical decisions made under uncertainty in dentistry, the role of social media in this process has not been sufficiently investigated [9]. Social media has revolutionized patient communication in pediatric dentistry yet ethical and regulatory frameworks are still absent [10]. Parents continue using these platforms despite expressing 72.7% skepticism about social media reliability [11]. Medical staff can use their understanding of these information-seeking behaviors to address parental misconceptions which leads to improved treatment adherence.

The primary research question of this study investigates how social media platforms affect parents’ dental treatment choices under general anesthesia and sedation through cognitive and emotional processes while educational level and health literacy influence these effects. This study investigates the types of social media content parents encounter while examining their criteria for evaluating trustworthiness and the impact of this information on their treatment choices. The study investigates both the occurrence of negative content exposure and its connection to social media usage duration and the impact of demographic characteristics on this process. The research results will help create educational programs for patients and communication strategies for doctors to engage with parents during pediatric dental anesthesia procedures.

## Method

### Data collection instruments

Some of the survey questions were developed by the authors for this study. It was originally created and administered in Turkish, and an English version was produced solely for publication purposes without undergoing a formal back-translation process. The researchers recognize this translation barrier as a research limitation. Content validity was assessed by one anesthesiologist and three dentists, each of whom independently rated the items using a 4-point Likert scale. Each item was evaluated using a four-point Likert scale (1 = not appropriate, 4 = completely appropriate). The researchers intentionally used a 4-point scale which excluded a neutral point to prevent participants from choosing neutral options, thereby requiring them to select a specific direction. The study design included a forced-choice format which was recognized as a limitation because it might not detect actual ambivalence among participants (see Limitations section). The Content Validity Index (CVI) was calculated by dividing the number of experts who rated each item as “appropriate” or “completely appropriate” by the total number of raters. Items with a CVI below 0.80 were revised accordingly. Based on their evaluations, the Content Validity Index (CVI) was calculated and found to be acceptable.

The questionnaire contained three main sections which included (1) independent items measuring separate aspects of clinical work and organizational environment but these items lacked internal consistency because they formed no unified scale and (2) a multi-item trustworthiness assessment scale for evaluating information sources with Cronbach’s alpha calculation and (3) open-ended questions that cannot be analyzed for internal consistency. The validity and reliability of the survey questions that do not belong to the authors (health literacy questions) in Turkish have been ensured.

### Study design and population

The research followed STROBE (Strengthening the Reporting of Observational Studies in Epidemiology) guidelines for conducting this cross-sectional descriptive study. The STROBE checklist serves its primary purpose for analytical observational studies which include cohort and case-control and cross-sectional analytical designs, but researchers now use it to enhance methodological and findings transparency in descriptive cross-sectional studies. The researchers used STROBE guidelines to improve their research methods and reporting quality because the EQUATOR Network supports better research reporting through these guidelines [12].

Parents of patients who received dental care at Necmettin Erbakan University Faculty of Dentistry Department of Paediatric Dentistry for routine treatment but could not receive clinic treatment and needed hospital sedation procedures were included in this study. The hospital operating room received patients for dental procedures under general anesthesia (GA) or deep sedation when they showed any of the following clinical signs: (1) The patient needed multiple dental procedures which exceeded what could be done during one standard clinic visit. (2) The patient was under three years old and unable to cooperate during treatment. (3) The patient had severe early childhood caries (S-ECC) which affected multiple teeth in their mouth. (4) The patient did not react to any behavioral treatment methods which included tell-show-do and voice control and protective stabilization during their previous clinic appointments. (5) The patient had special healthcare requirements which made them unable to follow standard treatment procedures. (6) The patient needed emergency surgery because their dental infection continued to be active. (7) The patient needed complete dental trauma treatment which required performing procedures in a controlled environment. The operating room environment became essential for these cases because it provided proper airway management and physiological monitoring and safe patient care during the extended dental procedures which standard dental clinics cannot support. General anesthesia is not indicated for patients who need single-tooth procedures because these procedures occur in outpatient clinics which do not use sedation. Data collection took place between May 2024 and September 2024.

The research excluded participants who were under 18 years old and parents who were not part of the study and those who provided duplicate responses and had more than 20% missing data in their answers. The research team used a formula from the literature to calculate *n* = 385 parents through non-probability/convenience sampling. The research findings may lose their general applicability because convenience sampling creates a limitation in the study.

### Variable definitions

The operational definitions of the variables used in the study were grouped into seven main categories:

1. The research includes three age groups ranging from 25 to 34, 35 to 44, and people who are 45 or older. The study examines three demographic groups based on gender and three educational levels which include primary school, high school, and university education. The research divides household income into three categories based on the 2024 national minimum wage which stands at 17,002 TL per month (Official Gazette December 30, 2023): below minimum wage (< 17,002 TL), 1–2 times minimum wage (17,002–34,004 TL), and above 2 times minimum wage (> 34,004 TL). The researchers used this system to create an appropriate socioeconomic classification which was suitable for Turkish society during the research period and to analyze children based on their age groups (under 3 years old, 3–6, 7–10).
2. The study examined two social media behavior patterns which included how often people used social media (less than one hour, one to three hours, or more than three hours) and which platform they used most often (Facebook/Instagram/YouTube/Twitter/Forums/Blogs). The research grouped Twitter with forums and blogs into one category because it combined three separate communication platforms which have different user behaviors and content patterns, although this combination was required because of low response rates for each platform alone. The research excluded TikTok from its response options because this platform had not gained sufficient popularity in Turkey at the time of data collection; researchers should include this platform in their future research. The research study did not include AI-based conversational agents (ChatGPT and Gemini) as information sources because these platforms have become vital health information resources despite their recent appearance. The study period saw the digital information landscape transform at such a fast pace that it became methodologically impractical to include these new resources in the research.
3. GA/sedation information search behavior: binary variable (yes/no) for information seeking and primary information source (search engine/social media group/friend-relative/healthcare professional recommendation). The category ‘healthcare professional recommendation’ includes referrals and advice from physicians, dentists, and other clinical staff, as well as information obtained from official healthcare institution social media accounts and websites. The current information source categories do not explicitly include private clinic/physician social media accounts as a separate option or AI conversational agents (e.g., ChatGPT, Gemini), which have become notable health information sources; these omissions are acknowledged as limitations of the questionnaire design.
4. Information trustworthiness assessment: A 4-point Likert scale measured trustworthiness criteria which consisted of source credibility, scientific references, user reviews, and recency. The ‘trustworthiness score’ emerged from the median calculation of these items. Exploratory factor analysis (EFA) was conducted to examine the dimensionality of the trustworthiness assessment scale. Principal axis factoring with Varimax rotation was employed. The analysis produced one factor which explained 52.4% of total variance with an eigenvalue of 2.10. The measurement scale yielded factor loadings which ranged from 0.61 for user reviews to 0.79 for source credibility, and all items exceeded the minimum required loading of 0.40.
5. Treatment decision influence: A 4-point scale assessed the impact of information on treatment choices (very influential/influential/partly influential/not influential). Two distinct categorical questions measured the changes in awareness and the shifts in risk perception.
6. Emotional and expectation measures: The visual analogue scale (VAS) with ratings from 1 to 10 evaluated anxiety, confidence, and satisfaction expectations before and after exposure to social media information. Social media authenticity (trust in posts) received a rating from 1 to 10.
7. Negative content exposure: The two dichotomous questions evaluated negative content exposure through yes/no response options. The content assessment included three categories which were complication narratives and misleading information and negative experiences. The restricted assessment method creates a research limitation but it shows first impressions about how people encounter negative content.

The health literacy score was determined by the Turkish HLS-SF12 and classified into three levels (tertile-based classification) according to the distribution: low, medium, and high. The Turkish HLS-SF12 shows confirmed validity and reliability in our nation according to the reported Cronbach’s alpha value of 0.856 [13]. The field of health benefits significantly from social media platforms because they enable users to share their experiences and maintain two-way interactions [14, 15].

### Study procedures

Research data were collected using a structured, three-part face-to-face questionnaire based on a literature review. The questionnaires were administered through one-on-one interviews with the parents of patients referred to the hospital operating room for dental treatment under general anesthesia or deep sedation at the Department of Paediatric Dentistry, Faculty of Dentistry, Necmettin Erbakan University.

The first section of the questionnaire inquired about the participants’ socio-demographic characteristics and social media usage habits. The second section included 4-point Likert-type statements regarding the impact of social media on the treatment decision-making process in the context of GA/sedation. The third section evaluated changes in anxiety, confidence, and satisfaction levels during the GA/sedation approach, institution selection, and pre- and post-treatment periods. The survey items were compiled by adapting scales and question stems from previous studies.

Instrument Validation: The content validity of the survey was ensured by expert opinion. The multi-item trustworthiness assessment scale demonstrated acceptable reliability through its Cronbach’s alpha coefficient which reached 0.78. A Cronbach’s alpha value ≥ 0.70 is considered acceptable, while a value between 0.80 and 1.00 is interpreted as high reliability [16]. KMO > 0.50 and the significance of Bartlett’s sphericity test were adopted as standard acceptance criteria in assessing the suitability of the scales for factor analysis [9]. The KMO value reached 0.742 while Bartlett’s test of sphericity produced a significant result (χ² = 486.3, df = 6, *p* < 0.001) which showed that the data were suitable for factor analysis. Subsequently, exploratory factor analysis was performed, and the results are reported in the Results section. The Turkish validity/reliability findings and index formula (Index = (Mean − 1)×50/3) of the HLS-SF12 were used as the basis for the classification in this study [13]. During field application, all responses were restricted to a single session, and duplicate entries were removed from the data set.

The use of Cronbach’s alpha for internal consistency in Likert-based items and the interpretation of threshold values are recommended in the methodology literature [16]. KMO and Bartlett tests are widely used and validated prerequisite tests for suitability for factor analysis [16]. The Turkish adaptation of the HLS-SF12 has been found suitable for the adult population in our country [13].

### Analytical framework

As this study is observational in nature, randomisation or experimental intervention was not applied. The subgroups to be used in the analyses were predefined and reported in the results tables. The defined subgroups are as follows: educational level (primary/high school/university and above), age group (25–34/35–44/≥45), household income level relative to minimum wage (below minimum wage/1–2× minimum wage/above 2× minimum wage), health literacy level (low/medium/high), social media usage time (< 1 h/1–3 h/>3 h), information seekers/non-seekers, and those exposed/not exposed to negative content.

Outcome variables related to GA/sedation were compared between these groups. The outcome variables compared included the level of influence on the treatment decision, pre- and post-anxiety, confidence and satisfaction scores, and social media authenticity scores. Types of negative content (complication narratives, false/misleading information, negative experiences) and the relationship between exposure and social media usage time were also described. Examining the impact of social media-derived information and experiences on parents’ GA/sedation decision-making processes is an approach recommended in the literature [17].

### Statistical analysis

Statistical analyses were performed using the SPSS 23 software package. The research team used Shapiro-Wilk tests together with visual inspection methods (histograms with normal curves and Q-Q plots) to study the distribution of continuous variables which included VAS measurements and Likert-derived composite scores. The Shapiro-Wilk test showed that all primary outcome variables had statistically significant deviations from a normal distribution (*p* < 0.05), which necessitated non-parametric statistical methods for analysis. Multi-item Likert-type scores that did not show a normal distribution were considered non-parametric. The Mann-Whitney U test was used to compare two independent groups, and the Kruskal-Wallis test was used to compare more than two independent groups. The chi-square test was used to analyse categorical data, and the Fisher Exact test was preferred when necessary. The Wilcoxon signed-rank test was applied to compare pre- and post-scores. Missing data were managed using the listwise deletion method.

Reporting rules were determined as follows: Given the non-normal distribution of continuous variables confirmed by normality testing, all continuous variables are presented as median with interquartile range (IQR), which is the appropriate descriptive measure for non-parametric data. P values were reported as two-tailed, specifying for each comparison which parameters and which groups were compared and which test was used. The research presents all P-values in a standardized format which includes two decimal places for values above 0.01 (e.g. P = 0.03), three decimal places for values between 0.001 and 0.01 (e.g. P = 0.008), and “P < 0.001” for values below 0.001. The significance level is accepted as α = 0.05. For Cronbach’s alpha interpretation thresholds, the range 0.60–0.79 is considered ‘reliable,’ and the range 0.80–1.00 is considered ‘high reliability’ [18]. The threshold values for KMO and Bartlett tests in factor analysis suitability are specified in the methodological literature [13].

### Ethical considerations

This study was approved by our institution’s Clinical Research Ethics Committee on 25.04.2024 with decision number: 2024/413. Written informed consent was obtained from all participants during face-to-face interviews. Informed consent to participate was obtained from the parents or legal guardians of all participants under the age of 16. Written informed consent for publication of clinical details and/or clinical images was obtained from the parents of all patients under the age of 18. Personal data were anonymised and coded for analysis, and data security was ensured through encrypted computers and databases accessible only to the study team. The study was conducted in accordance with the principles of the Helsinki Declaration and Good Clinical Practice. The study provides detailed anonymized case evaluation data which includes diagnoses, procedures, anesthesia types, ASA classifications, and treatment results in Supplementary Tables S1 and S2.

## Results

The characteristics of the 385 parents who participated in the study and their social media usage are presented in Table 1. Of the total 385 participants, 308 (80.0%) actively sought information about GA/sedation on social media platforms and were included in the subsequent analyses related to information-seeking behaviors. The remaining 77 participants (20.0%) did not search for GA/sedation information on social media. The 17,002 TL net minimum wage of 2024 serves as a reference point to study how different income categories distribute their household earnings. The study results indicate that 93 out of 385 participants (24.2%) earned less than the minimum wage while 167 out of 385 participants (43.4%) earned between 1 and 2 times the minimum wage and 125 out of 385 participants (32.4%) earned more than twice the minimum wage. Looking at the age distribution of the children, 90/385 (23.4%) were under 3 years old, 171/385 (44.4%) were 3–6 years old, and 124/385 (32.2%) were in the 7–10 age group. Health literacy was assessed using the HLS-SF12 scale, and the median index score was found to be 38.0 (IQR: 32.0–44.0). The Shapiro-Wilk test indicated that the HLS-SF12 scores were not normally distributed (W = 0.967, *p* < 0.001), justifying the use of non-parametric tests. Classification based on tertile values showed that 128/385 (33.2%) of participants had low (≤ 33 points), 129/385 (33.5%) had medium (34–42 points), and 128/385 (33.3%) had high (≥ 43 points) health literacy levels (Table 1)**.**

**Table 1:** Participant characteristics and social media use (*n* = 385)

Characteristic	*n*	%
Socio-demographic Characteristics		
Age Groups		
25–34 years	112	29.1
35–44 years	169	43.9
≥45 years	104	27.0
Gender		
Female	246	63.9
Male	139	36.1
Educational Status		
Primary education	62	16.1
High school	108	28.1
University or higher	215	55.8
Household Monthly Income*		
< Minimum wage (< 17,002 TL)	93	24.2
1–2× Minimum wage (17,002–34,004 TL)	167	43.4
> 2× Minimum wage (> 34,004 TL)	125	32.4
Child’s Age		
<3 years	90	23.4
3–6 years	171	44.4
7–10 years	124	32.2
Social Media Use Characteristics		
Daily Usage Time		
<1 h	50	13.0
1–3 h	192	49.9
>3 h	143	37.1
Most Frequently Used Platform		
Facebook	138	35.8
Instagram	123	32.0
YouTube	77	20.0
Twitter/Forums/Blogs	47	12.2
Health Literacy (HLS-SF12)		
Low (≤ 33 points)	128	33.2
Moderate (34–42 points)	129	33.5
High (≥ 43 points)	128	33.3
HLS-SF12 Index Score, Median (IQR)	38.0 (32.0–44.0)	

*HLS-SF12* Short-Form Health Literacy Scale (12 items), *IQR* Interquartile Range

*Income categories based on 2024 Turkish net minimum wage (17,002 TL/month; Official Gazette, December 30, 2023). Shapiro-Wilk test for HLS-SF12: W = 0.967, *p* < 0.001

The internal consistency of the trustworthiness assessment criteria was found to be Cronbach’s alpha = 0.78, and it was determined to be of an acceptable level of reliability. Exploratory factor analysis (EFA) was conducted using principal axis factoring with Varimax rotation. The KMO value for suitability for factor analysis was 0.742, and Bartlett’s sphericity test was found to be significant (χ²=486.3, *p* < 0.001), indicating that the scale was suitable for factor analysis. The EFA revealed a single-factor solution explaining 52.4% of the total variance. Factor loadings ranged from 0.61 to 0.79, with all items loading significantly on the single factor (Table 2). The median total trustworthiness score was 3.15 (IQR: 2.68–3.58) (Table 2, Table 3).

**Table 2:** Exploratory factor analysis results for trustworthiness assessment scale (*n* = 308)

Item	Factor Loading	Communality
Source credibility	0.79	0.62
Scientific references	0.76	0.58
User reviews	0.61	0.37
Recency of publication	0.68	0.46
Eigenvalue	2.10	
Variance Explained (%)	52.4	

Extraction method: Principal Axis Factoring. Rotation method: Varimax with Kaiser Normalization. KMO = 0.742; Bartlett’s Test of Sphericity: χ² = 486.3, df = 6, *p* < 0.001. Factor loadings ≥ 0.40 considered significant

**Table 3:** GA/Sedation Information-Seeking behaviors and trustworthiness assessment

Variable	*n*/*N* (%)	Median (IQR)	Cronbach’s α
Information-Seeking Status (*n* = 385)		–	–
Sought GA/Sedation information	308/385 (80.0)	–	–
Did not seek information	77/385 (20.0)	–	–
First Information Source (*n* = 308)		–	–
Search engines	105/308 (34.1)	–	–
Social media groups	83/308 (26.9)	–	–
Friends/relatives	68/308 (22.1)	–	–
Physician referral	52/308 (16.9)	–	–
Trustworthiness Assessment (*n* = 308)			0.78
Source credibility	265/308 (86.0)	3.50 (3.00–4.00)	
Scientific references	251/308 (81.5)	3.25 (2.75–3.75)	
User reviews	196/308 (63.6)	2.75 (2.00–3.50)	
Recency of publication	234/308 (76.0)	3.00 (2.50–3.50)	
Total Trustworthiness Score	–	3.15 (2.68–3.58)	

Continuous variables are reported as Median (IQR) due to non-normal distribution (Shapiro-Wilk *p* < 0.05). Cronbach’s α indicates internal consistency

*GA *General Anesthesia, *IQR* Interquartile Range

The impact on treatment decisions and emotional changes are shown in Table 4. Social media authenticity was defined as the level of trust parents felt in social media posts and was measured with a median of 7.00 (IQR: 5.50–8.00). Among the 308 information seekers, 199/308 (64.6%) reported that social media information influenced their treatment decision (very influential: 68/308, 22.1%; influential: 131/308, 42.5%). When institutional preferences were examined, 142/308 (46.1%) preferred private hospitals, 89/308 (28.9%) preferred public hospitals, and 77/308 (25.0%) preferred university hospitals. Emotional state changes were analysed using the Wilcoxon signed-rank test (Table 4).

**Table 4:** Influence on treatment Decisions, emotional Changes, and institutional preferences (*n* = 308)

Variable	*n* (%)	Before Median (IQR)	After Median (IQR)	*p*-value*
Influence on Treatment Decision				
Very influential	68 (22.1)	–	–	
Influential	131 (42.5)	–	–	
Partly influential	80 (26.0)	–	–	
Not influential	29 (9.4)	–	–	
Influence score	–	–	3.50 (2.75–4.00)	
Emotional Changes				
Anxiety level (1–10)	–	7.00 (6.00–8.50)	6.00 (4.50–7.50)	< 0.001
Confidence level (1–10)	–	5.50 (4.00–7.00)	7.00 (5.50–8.00)	< 0.001
Satisfaction expectation (1–10)	–	6.50 (5.00–7.50)	7.00 (6.00–8.00)	0.008
Social Media Familiarity				
Trust in posts (1–10)	–	–	7.00 (5.50–8.00)	–
Institutional Preference				
Private hospital	142 (46.1)	–	–	
Public hospital	89 (28.9)	–	–	
University hospital	77 (25.0)	–	–	

*IQR* Interquartile Range

*Wilcoxon signed-rank test applied for paired comparisons. Continuous variables reported as Median (IQR) consistent with non-parametric analysis

The effect of demographic factors is presented in Table 5. When examined in terms of income status, the trustworthiness score (median 3.50, IQR: 3.00–4.00) and information application skills (median 4.00, IQR: 3.50–4.75) of the group whose income was greater than 2× minimum wage were found to be significantly higher than those of the group whose income was below minimum wage (median 3.00, IQR: 2.25–3.50 and median 2.50, IQR: 1.75–3.00, respectively) (*p* < 0.001). Categorical variables were analysed using the chi-square test, while group comparisons of continuous variables were performed using the Kruskal-Wallis test. The Mann-Whitney U test was used for pairwise comparisons **(**Table 5**).**

**Table 5:** Effects of demographic factors on information seeking and treatment decision

Variable	Information Seeking *n*/*N* (%)	Trustworthiness Score Median (IQR)	Treatment Decision Influence Median (IQR)	Information Application Median (IQR)
Educational Status				
Primary education	48/62 (77.4)	2.75 (2.00–3.50)	3.75 (3.00–4.50)	2.25 (1.50–3.00)
High school	85/108 (78.7)	3.25 (2.50–3.75)	3.50 (2.75–4.25)	3.25 (2.50–4.00)
University+	175/215 (81.4)	3.75 (3.00–4.25)	3.25 (2.50–4.00)	4.25 (3.75–4.75)
*p*-value	0.624ᵃ	< 0.001ᵇ	0.021ᵇ	< 0.001ᵇ
Age Groups				
25–34 years	92/112 (82.1)	3.50 (3.00–4.25)	3.75 (3.00–4.50)	4.00 (3.25–4.75)
35–44 years	135/169 (79.9)	3.50 (2.75–4.00)	3.50 (2.50–4.00)	3.50 (2.75–4.25)
≥45 years	81/104 (77.9)	3.25 (2.50–3.75)	3.25 (2.25–4.00)	3.00 (2.25–3.75)
*p*-value	0.581ᵃ	0.038ᵇ	0.042ᵇ	0.016ᵇ
Household Income*				
< Min. wage	71/93 (76.3)	3.00 (2.25–3.50)	3.75 (3.00–4.50)	2.50 (1.75–3.00)
1–2× Min. wage	133/167 (79.6)	3.25 (2.50–3.75)	3.50 (2.75–4.00)	3.50 (2.75–4.00)
> 2× Min. wage	104/125 (83.2)	3.50 (3.00–4.00)	3.25 (2.50–4.00)	4.00 (3.50–4.75)
*p*-value	0.268ᵃ	< 0.001ᵇ	0.035ᵇ	< 0.001ᵇ

*IQR* Interquartile Range, *Min. wage* Minimum wage

ᵃChi-square test. ᵇKruskal–Wallis test. *Based on 2024 Turkish net minimum wage (17,002 TL/month). All continuous variables reported as Median (IQR) consistent with non-parametric statistical tests

Negative content exposure is detailed in Table 6. The distribution of social media usage time among the groups was evaluated among 308 information seekers: those using it less than 1 h (*n* = 40), those using it 1–3 h (*n* = 154), and those using it 3 + hours (*n* = 114). The exposure rates within these groups were compared using the chi-square test, and a significant difference was found (*p* = 0.034) (Table 6).

**Table 6:** Relationship between exposure to negative content and duration of social media use

Variable	*n*	%
Exposure to Negative Content/Experience (*n* = 308)		
Yes	185	60.1
No	123	39.9
Social Media Use Duration and Exposure		
< 1 h (*n* = 40)		
– Exposed	18	45.0
– Not exposed	22	55.0
1–3 h (*n* = 154)		
– Exposed	91	59.1
– Not exposed	63	40.9
> 3 h (*n* = 114)		
– Exposed	76	66.7
– Not exposed	38	33.3
*p*-value (Chi-square test)	–	0.034

Exposure comparisons evaluated using Chi-square test

## Discussion

This study aimed to examine the social media usage habits of parents whose children were scheduled to undergo dental treatment under general anaesthesia or sedation, and the impact of these platforms on treatment decisions. Our findings reveal that the vast majority of parents in the modern age actively use social media as a source of information prior to making medical decisions, and that this has both positive and negative implications. In particular, it was observed that content encountered on digital platforms significantly shapes parents’ emotional states, risk perceptions, and ultimately their treatment preferences. Educational level and health literacy were found to play a critical role in this process; however, interestingly, parents with lower levels of education were found to be more influenced by social media content. The research findings demonstrated that parents base their decisions about pediatric dental anesthesia treatment on their social media beliefs which influence their willingness to give consent and their children’s appointment attendance.

Our findings show that 80% of parents use social media to search for information about GA/sedation and 37.1% use social media for more than 3 h a day. This high usage rate supports the notion that social media is an important source of information among parents. Griauzde et al. reported that 75% of Hispanic mothers use social media at least once a day and that few mothers reported social media influencing their feeding decisions [19]. This is partially consistent with our findings, showing similarities in terms of widespread social media use, although our study observed more intensive information-seeking behaviour. The preference for search engines (34.1%) and social media groups (26.9%) as the primary information source parallels Kubb and Foran’s finding that parents frequently use search engines to find online health information for their children; however, interest in social media groups was more pronounced in our study [3]. The three most popular platforms used by parents are Facebook (35.8%), Instagram (32.0%) and YouTube (20.0%) which confirms Jaks et al. that parents use different digital media platforms [20]. Frey’s research on parental use of social media for health information acquisition supports the findings of our study [21]. Healthcare providers who understand platform preferences can create individualized educational programs for their patients through their knowledge of these preferences.

Our findings show that university graduates have a higher trustworthiness score (median 3.75, IQR: 3.00–4.25) than primary school graduates (median 2.75, IQR: 2.00–3.50) and that this difference is statistically significant (*p* < 0.001). This supports the effect of educational level on information evaluation and is consistent with findings by Rosenblatt et al. that parents do not receive adequate education in anaesthesia-related decision-making processes, implying that the ability to evaluate information may be related to education [22]. However, the fact that the effect on treatment decisions was greater among primary school graduates (median 3.75, IQR: 3.00–4.50) than among university graduates (median 3.25, IQR: 2.50–4.00) (*p* = 0.021) presents a notable contradiction. The paradox exists because of multiple reasons. People with lower education levels tend to lack critical thinking abilities which makes them more likely to believe information without question. People who have lower education levels tend to trust social media testimonials and peer experiences over scientific evidence because these formats are easier to understand and create stronger emotional connections. The Turkish cultural environment makes less educated parents trust community-based information sources above expert opinions because they find shared personal experiences more important than professional advice. The clinical findings show that dental practitioners should develop chairside communication systems which are aligned with parental educational levels because they must address social media-based false information when treating parents who have lower educational attainment.

Malone et al. suggest that parents’ information-seeking behaviour is influenced by the social and cultural environment and that this may be shaped by preferences independent of educational level [23]. The finding that information application skills increase with education (primary school median 2.25, IQR: 1.50–3.00; university median 4.25, IQR: 3.75–4.75, *p* < 0.001) is consistent with the findings of Kubb and Foran that parents need guidance when searching for online health information; emphasising the critical role of educational level in applying knowledge [3]. A similar relationship was also observed in terms of income status, with the group earning above 2× minimum wage having significantly higher trustworthiness scores (median 3.50, IQR: 3.00–4.00) and information application skills (median 4.00, IQR: 3.50–4.75) than those of the group earning below minimum wage (median 3.00, IQR: 2.25–3.50 and median 2.50, IQR: 1.75–3.00, respectively) (*p* < 0.001).

Our research shows that 60.1% of parents encountered negative content on social media. Hairston et al.‘s study specifically examining parental concerns about pediatric tonsillectomy shared on social media platforms reported that parents shared their fears and concerns about tonsillectomy on social media and that this influenced their decision-making processes; this is consistent with our findings and supports the impact of negative content [23]. Furthermore, the finding that exposure was 66.7% among those using social media for 3 + hours and 45.0% among those using it for < 1 h (*p* = 0.034) parallels Patrick et al.‘s analysis of social media’s role in healthcare decision-making, which suggests that social media may influence health decisions and that the spread of misinformation may be related to usage time [23, 24]. The fact that exposure to negative content increases as social media usage time increases suggests that time spent on digital platforms may be a risk factor. Djalali Talab and Geibel’s investigation of parental acceptance of dental treatment under general anesthesia noted that parents expressed fears about GA and that this shaped their decisions, confirming the potential impact of exposure to negative content on treatment decisions in our findings [25, 26]. The research findings demonstrate that healthcare providers should actively address false information which parents discover through social media during their preoperative meetings because these parents tend to find such content online.

Menezes et al.‘s systematic review of online health information’s impact on pediatric treatment decisions also supports the findings of our study [27]. Our findings show that social media information was associated with treatment decisions (median influence score 3.50, IQR: 2.75–4.00) and that the emotional effects of social media play an important role in the decision-making process.

64.6% of participants (199/308) reported that social media information influenced their treatment decision (very influential 22.1%, influential 42.5%). Ma et al.‘s research on family anxiety and pediatric procedural preparation reported that family members’ anxiety levels were positively correlated with children’s anxiety and that information provision methods reduced anxiety; this is consistent with our finding that anxiety levels were associated with a decrease from median 7.00 (IQR: 6.00–8.50) to 6.00 (IQR: 4.50–7.50) (*p* < 0.001) [28]. The increase in confidence levels from median 5.50 (IQR: 4.00–7.00) to 7.00 (IQR: 5.50–8.00) (*p* < 0.001) and satisfaction expectations from median 6.50 (IQR: 5.00–7.50) to 7.00 (IQR: 6.00–8.00**)** (*p* = 0.008) are supported by Kakti et al.‘s study on factors influencing parental acceptance of dental treatment under general anesthesia, indicating that parental acceptance of general anaesthesia increases with knowledge [29]. Merino et al.‘s analysis of social media’s impact on health perceptions expressed that social media can shape individuals’ perceptions both positively and negatively, which explains the dual effects on the decision-making process [30]. Abdelaziz’s evaluation of educational video effectiveness for parental GA preparation found parents’ positive responses to general anaesthesia information videos, supporting that the information sources in our study were associated with parental attitudes [31]. Medical staff need to understand emotional changes in patients because these changes help patients feel less anxious while their parents gain confidence which results in better cooperation during consent procedures and enhanced preoperative and postoperative medical order compliance.

Clinical Implications: The research results from this study provide clinicians who treat pediatric dental anesthesia patients with multiple practical treatment methods for their patients. Clinicians need to ask parents about their social media information-seeking activities during preoperative consultations to detect and address any misconceptions they might have. Healthcare institutions need to create evidence-based social media content which will deliver accurate information about GA/sedation procedures to patients. The development of patient education materials requires tailored content for parents who have different levels of education because basic information should reach parents who have limited education and who might believe false information. Clinicians need to provide parents who show excessive social media behavior and negative online content exposure with additional consultation time to address their specific requirements. The strategies outlined will enhance patient willingness to receive treatment while reducing the number of patients who cancel their appointments without warning, which will result in improved medical outcomes.

This study has several limitations that should be acknowledged. The study design prevents us from determining cause-and-effect relationships because it uses a cross-sectional approach and the participants came from a single provincial center. The selection bias in convenience sampling occurs because parents who joined the study differ from those who refused to participate. The self-report bias affects the study because parents might provide inaccurate information about their social media usage and its impact on their choices because they want to appear socially acceptable. The study results become less comparable across international settings because the questionnaire transition from Turkish to English occurred without proper back-translation procedures. The assessment of negative content exposure through two simple yes or no questions creates a methodological limitation that fails to represent the complete nature of this construct. The study contains a methodological limitation because it uses a 4-point Likert scale which lacks a neutral point. The researchers chose this forced-choice format to prevent neutral responses while requiring respondents to choose between two options, but this method might not fully capture how uncertain or ambivalent participants really are. Research studies may benefit from using 5-point or 7-point rating systems which include a middle point to obtain more nuanced responses from participants. The social media platform categories studied in this research contain specific constraints which affect their analysis. The combination of Twitter with forums and blogs into one category unites three separate communication spaces which have different user behaviors and content patterns, which might obscure essential variations in how users search for information between these platforms. The survey did not include TikTok as an answer choice because this platform has gained substantial usage among Turkish users who belong to the younger age group. The list of information sources did not include AI-based conversational agents which include ChatGPT and Gemini, although these tools have become increasingly important sources for health information. The research acknowledges the need to incorporate these absent data points because digital information undergoes rapid transformations in the contemporary digital environment. The study achieves its strengths through its wide participant range and complete assessment of social media effects on parental choices. The research needs expansion to multiple locations and should track parental decision-making changes through time using longitudinal methods. The development of intervention programs requires assessment methods to verify social media health information accuracy while teaching parents how to identify trustworthy sources.

## Conclusion

Social media platforms were reported as a primary information resource for parents who decide between general anesthesia and sedation for their children’s dental procedures. These platforms were associated with shaping their treatment choices. The study found that social media content was associated with increased parental confidence and decreased anxiety levels. The study shows that negative content exposure occurred frequently among participants but it does not prove that such content leads to treatment avoidance. The research shows that people with better education and health literacy skills evaluate digital content better. The less educated population was found to be more vulnerable to social media influence according to the study results. The clinical results demonstrate that pediatric dentists and anesthesiologists must take an active role in addressing digital information which affects their practice. Healthcare professionals must increase their digital platform presence to help parents find reliable information sources. Medical staff need to check parental social media activity during preoperative meetings through established procedures which they should use to fix any incorrect information. The study design as a cross-sectional analysis shows associations between variables instead of proving direct cause-and-effect relationships. Research studies following participants over time should be conducted to determine cause-and-effect relationships and monitor how parental choices evolve. Research should evaluate the techniques which clinicians who operate on social media platforms need to use to enhance parent understanding and dental anesthesia acceptance for pediatric dental procedures.

## Supplementary Information

Supplementary Material 1.

Supplementary Material 2.

Supplementary Material 3.

## Data Availability

Data used in this study can be provided upon reasonable request.

## Abbreviations

GA: General Anesthesia
HLS-SF12: Health Literacy Scale-Short Form 12
SPSS: Statistical Package for the Social Sciences
KMO: Kaiser-Meyer-Olkin
SD: Standard Deviation
SE: Standard Error
SEM: Standard Error of the Mean

## References

[1] Hulin J, Baker SR, Marshman Z, et al. Development of a decision aid for children faced with the decision to undergo dental treatment with sedation or general anaesthesia. Int J Paediatr Dent. 2017;27(5):344–55. 10.1111/ipd.12267. 27684707 10.1111/ipd.12267

[2] Zhang X, Fan Z, He D, Liu Y, Shi X, Zhang H. Effectiveness of dexmedetomidine as a premedication for pediatric patients undergoing outpatient dental surgery under general anaethesia-systematic review and meta-analysis. PeerJ. 2025;13:e19216. 10.7717/peerj.19216. 40183044 10.7717/peerj.19216 PMC11967424

[3] Kubb C, Foran HM. Online health information seeking by parents for their children: systematic review and agenda for further research. J Med Internet Res. 2020;22(8):e19985. 10.2196/19985. 32840484 10.2196/19985 PMC7479585

[4] Sasikala D, Norouzi Baghkomeh P, Farzan JM, et al. Use of social media by parents as a resource for knowledge on children’s oral health: a systematic review. Can J Dent Hyg. 2025;59(1):45–58. 40170949 PMC11961065

[5] Turkish Statistical Institute. Survey on Information and Communication Technology (ICT) Usage in Households and by Individuals. 2024. Release date: 27 August 2024. Publication No: 53492. Available from: https://data.tuik.gov.tr/Bulten/Index?p=Survey-on-Information-and-Communication-Technology-(ICT)-Usage-in-Households-and-by-Individuals-2024-53492&dil=2 (Accessed: [18.10.2025]).

[6] Gürün S, İkikat Tümer E. The effect of social media influencers and advertising on the online shopping behavior of different generations. Reforma. 2025;(101):24–32.

[7] Zysset AE, Schwärzler P, Dratva J. Seeking health in a digital world: exploring immigrant parents’ quest for child health Information—A scoping review. Int J Environ Res Public Health. 2023;20(19):6804. 10.3390/ijerph20196804. 37835074 10.3390/ijerph20196804 PMC10572919

[8] Coughler C, Burke M, Cardy S. Analysis of the quality of online resources for parents of children who are late to talk. Autism Dev Lang Impair. 2020;5:2396941520917940. 10.1177/2396941520917940. Published 2020 Apr 6. 36381543 10.1177/2396941520917940 PMC9620457

[9] Murdoch AIK, Blum J, Chen J, Baziotis-Kalfas D, Dao A, Bai K, Bekheet M, Atwal N, Cho SSH, Ganhewa M, et al. Determinants of clinical decision making under uncertainty in dentistry: A scoping review. Diagnostics. 2023;13(6):1076. 10.3390/diagnostics13061076. 36980383 10.3390/diagnostics13061076 PMC10047498

[10] Bhatara S, Goswami M, Saxena A, et al. The evolving role of social media in paediatric dentistry: a narrative review. Glob Pediatr. 2024;9:100221. 10.1016/j.gpeds.2024.100221.

[11] Krishnan GA, Nair AK, Bhaskar BV. Comprehensive analysis of factors influencing patients’ preferences and attitudes toward dental treatment choices. Ann Afr Med. 2025;24:237–43. 10.4103/aam.aam_125_24. 39787352 10.4103/aam.aam_125_24 PMC12103110

[12] von Elm E, Altman DG, Egger M, et al. The strengthening the reporting of observational studies in epidemiology (STROBE) statement: guidelines for reporting observational studies. Lancet. 2007;370(9596):1453–7. 10.1016/S0140-6736(07)61602-X. 18064739 10.1016/S0140-6736(07)61602-X

[13] Karahan Yılmaz S, Eskici G. Sağlık Okuryazarlığı Ölçeği-Kısa Form ve Dijital Sağlıklı Diyet Okuryazarlığı Ölçeğinin Türkçe Formunun Geçerlik ve Güvenirlik Çalışması. İzmir Kâtip Çelebi Üniv Sağlık Bil Fak Derg. 2021;6(3):19–25.

[14] Antheunis ML, Tates K, Nieboer TE. Patients’ and health professionals’ use of social media in health care. Patient Educ Couns. 2013;92(3):426–31. 23899831 10.1016/j.pec.2013.06.020

[15] Korda H, Itani Z. Harnessing social media for health promotion and behavior change. Health Promot Pract. 2013;14(1):15–23. 21558472 10.1177/1524839911405850

[16] Tabuk ME. Spor Bilimleri Alanında Yayımlanan Ölçeklerin Sistematik Analizi. CBÜ Beden Eğitimi ve Spor Bilimleri Dergisi. 2023;18(2):583–94. 10.33459/cbubesbd.1315816.

[17] Griffis HM, Kilaru AS, Werner RM, et al. Use of social media across US hospitals: descriptive analysis of adoption and utilization. J Med Internet Res. 2014;16(11):e264. 10.2196/jmir.3758. 25431831 10.2196/jmir.3758 PMC4260061

[18] Taber KS. The use of cronbach’s alpha when developing and reporting research instruments in science education. Res Sci Educ. 2018;48(6):1273–96. 10.1007/s11165-016-9602-2.

[19] Griauzde DH, et al. The influence of social media on child feeding practices and beliefs among Hispanic mothers: a mixed methods study. Eat Behav. 2020;36:101361. 31923649 10.1016/j.eatbeh.2019.101361 PMC8005295

[20] Jaks R, et al. Parental digital health information seeking behavior in Switzerland: a cross-sectional study. BMC Public Health. 2019;19:225. 30791927 10.1186/s12889-019-6524-8 PMC6385444

[21] Frey EFJ. Parents’ use of social media for health information: an Australian mixed methods study. University of Technology Sydney; 2024.

[22] Rosenblatt A et al. Parental Decision-Making for surgery and anesthesia in young children. West J Nurs Res. 2021. 10.1177/01939459211021622 34085888

[23] Malone M, et al. Parental health information seeking and re-exploration of the ‘digital divide’. Prim Health Care Res Dev. 2014;15:202–12. 23676618 10.1017/S1463423613000194

[24] Hairston TK, et al. Evaluation of parental perspectives and concerns about pediatric tonsillectomy in social media. JAMA Otolaryngol Head Neck Surg. 2019. 10.1001/jamaoto.2018.2917. 30452510 10.1001/jamaoto.2018.2917 PMC6439813

[25] Patrick M, et al. Social media and its impact on health care. Ann Allergy Asthma Immunol. 2022;128:139–45. 34555532 10.1016/j.anai.2021.09.014

[26] Djalali Talab Y, Geibel M. Comparison of parental and practitioner’s acceptance for dental treatment under general anaesthesia in paediatric patients. BMC Pediatr. 2023;23:45. 36707845 10.1186/s12887-022-03805-1 PMC9883120

[27] Menezes JDDS et al. Online health information and pediatric treatment decisions: A study of generation Y parents. Acta Biomed. 2024.

[28] Ma X, et al. Impact of pediatric surgery on anxiety in children and their families and coping strategies: a narrative review. Translational Pediatr. 2025;14(4):718–27. 10.21037/tp-2025-10 PMC12079698 40386357

[29] Kakti A, et al. Commonly reported factors influencing the parent’s decision to accept dental treatment under general anesthesia for their children. Med Sci. 2020;24(106):4808–12.

[30] Merino M, et al. Body perceptions and psychological well-being: a review of the impact of social media and physical measurements on self-esteem and mental health with a focus on body image satisfaction and its relationship with cultural and gender factors. Healthcare. 2024;12:1396. 39057539 10.3390/healthcare12141396 PMC11276240

[31] Abdelaziz A. Effectiveness of a Pre-Operative general anesthesia educational video on parent’s knowledge and anxiety. University of Illinois at Chicago; 2024.

