# Supplementary material for: The role of social media in parents’ approaches to dental treatment procedures under general anesthesia and sedation: a cross-sectional survey in Turkey
Source: BMC Oral Health. 2026 Feb 2;26:409. doi: 10.1186/s12903-026-07779-9 (PMC12952005; doi:10.1186/s12903-026-07779-9)
Supplement: Supplementary file 1 — Supplementary Material 1. [file 12903_2026_7779_MOESM1_ESM.pdf]

Here is the academic translation of your survey questions:

1. Sociodemographic Characteristics

* What is your age?

a. 25-34

b. 35-44

c. 45 and over

* What is your gender?

a. Female

b. Male

* What is your educational background?

a. Primary/Secondary School

b. High School

c. University or higher

* What is your monthly household income?

a. 10,000 TL or less

b. 10,001 TL - 20,000 TL

c. 20,001 TL or more

* What is the age of your child?

a. 3-6

b. 7-10

c. 11-14

2. Social Media Usage

* How many hours do you use social media on an average day?

a. Less than 1 hour

b. 1-3 hours

c. More than 3 hours

* Which social media platforms do you use most frequently? (You may select more than one option)

a. Facebook

b. Instagram

c. YouTube

d. Twitter, forums, or blogs

3. Information Seeking and Sources

* Have you searched for information about general anesthesia (GA) or sedation for your child via social media or the internet?

a. Yes

b. No

* What was your initial source of information on this topic?

a. Search engines (Google, Bing, etc.)

b. Social media groups

c. Friends or relatives

d. I benefited from the advice of another physician
