# Supplementary material for: The role of social media in parents’ approaches to dental treatment procedures under general anesthesia and sedation: a cross-sectional survey in Turkey
Source: BMC Oral Health. 2026 Feb 2;26:409. doi: 10.1186/s12903-026-07779-9 (PMC12952005; doi:10.1186/s12903-026-07779-9)
Supplement: Supplementary file 2 — Supplementary Material 2. [file 12903_2026_7779_MOESM2_ESM.docx]

**SUPPLEMENTARY MATERIAL**

**Supplementary Table S1. Anonymized Case Evaluation Portfolio**

| **Case ID** | **Age Group (years)** | **Gender** | **Primary Diagnosis** | **Procedure Performed** | **Anesthesia Type** | **ASA Class** | **Outcome** |
| --- | --- | --- | --- | --- | --- | --- | --- |
| P001 | <3 | F | Severe ECC | Multiple extractions + restorations | GA | ASA I | Successful |
| P002 | <3 | F | Multiple carious lesions | Pulpotomy + SSC + restorations | GA | ASA I | Successful |
| P003 | <3 | M | Dental trauma | Multiple extractions | GA | ASA I | Successful |
| P004 | <3 | F | Pulpal involvement | Restorations + pulpotomy | GA | ASA II | Successful |
| P005 | <3 | F | Early childhood caries | Full mouth rehabilitation | GA | ASA I | Successful |
| P006 | <3 | M | Extensive decay | Extractions + space maintainer | GA | ASA II | Successful |
| P007 | <3 | F | Failed behavioral management | Multiple SSC + restorations | GA | ASA I | Successful |
| P008 | <3 | F | Special healthcare needs | Pulpectomy + restorations | Deep Sedation | ASA I | Successful |
| P009 | <3 | M | Acute dental infection | Incision & drainage + extractions | GA | ASA II | Minor complication - resolved |
| P010 | <3 | F | Multiple teeth requiring extraction | Restorations only | GA | ASA I | Successful |
| P011 | <3 | F | Severe ECC | Multiple extractions + restorations | GA | ASA I | Successful |
| P012 | <3 | M | Multiple carious lesions | Pulpotomy + SSC + restorations | GA | ASA I | Successful |
| P013 | 3-6 | F | Dental trauma | Multiple extractions | GA | ASA I | Successful |
| P014 | 3-6 | F | Pulpal involvement | Restorations + pulpotomy | GA | ASA II | Successful |
| P015 | 3-6 | M | Early childhood caries | Full mouth rehabilitation | GA | ASA I | Successful |
| P016 | 3-6 | F | Extensive decay | Extractions + space maintainer | Deep Sedation | ASA II | Successful |
| P017 | 3-6 | F | Failed behavioral management | Multiple SSC + restorations | GA | ASA I | Successful |
| P018 | 3-6 | M | Special healthcare needs | Pulpectomy + restorations | GA | ASA I | Successful |
| P019 | 3-6 | F | Acute dental infection | Incision & drainage + extractions | GA | ASA II | Minor complication - resolved |
| P020 | 3-6 | F | Multiple teeth requiring extraction | Restorations only | GA | ASA I | Successful |
| P021 | 3-6 | M | Severe ECC | Multiple extractions + restorations | GA | ASA I | Successful |
| P022 | 3-6 | F | Multiple carious lesions | Pulpotomy + SSC + restorations | GA | ASA I | Successful |
| P023 | 3-6 | F | Dental trauma | Multiple extractions | GA | ASA I | Successful |
| P024 | 3-6 | M | Pulpal involvement | Restorations + pulpotomy | Deep Sedation | ASA II | Successful |
| P025 | 3-6 | F | Early childhood caries | Full mouth rehabilitation | GA | ASA I | Successful |
| P026 | 3-6 | F | Extensive decay | Extractions + space maintainer | GA | ASA II | Successful |
| P027 | 3-6 | M | Failed behavioral management | Multiple SSC + restorations | GA | ASA I | Successful |
| P028 | 3-6 | F | Special healthcare needs | Pulpectomy + restorations | GA | ASA I | Successful |
| P029 | 3-6 | F | Acute dental infection | Incision & drainage + extractions | GA | ASA II | Minor complication - resolved |
| P030 | 3-6 | M | Multiple teeth requiring extraction | Restorations only | GA | ASA I | Successful |
| P031 | 3-6 | F | Severe ECC | Multiple extractions + restorations | GA | ASA I | Successful |
| P032 | 3-6 | F | Multiple carious lesions | Pulpotomy + SSC + restorations | Deep Sedation | ASA I | Successful |
| P033 | 3-6 | M | Dental trauma | Multiple extractions | GA | ASA I | Successful |
| P034 | 3-6 | F | Pulpal involvement | Restorations + pulpotomy | GA | ASA II | Successful |
| P035 | 7-10 | F | Early childhood caries | Full mouth rehabilitation | GA | ASA I | Successful |
| P036 | 7-10 | M | Extensive decay | Extractions + space maintainer | GA | ASA II | Successful |
| P037 | 7-10 | F | Failed behavioral management | Multiple SSC + restorations | GA | ASA I | Successful |
| P038 | 7-10 | F | Special healthcare needs | Pulpectomy + restorations | GA | ASA I | Successful |
| P039 | 7-10 | M | Acute dental infection | Incision & drainage + extractions | GA | ASA II | Minor complication - resolved |
| P040 | 7-10 | F | Multiple teeth requiring extraction | Restorations only | Deep Sedation | ASA I | Successful |
| P041 | 7-10 | F | Severe ECC | Multiple extractions + restorations | GA | ASA I | Successful |
| P042 | 7-10 | M | Multiple carious lesions | Pulpotomy + SSC + restorations | GA | ASA I | Successful |
| P043 | 7-10 | F | Dental trauma | Multiple extractions | GA | ASA I | Successful |
| P044 | 7-10 | F | Pulpal involvement | Restorations + pulpotomy | GA | ASA II | Successful |
| P045 | 7-10 | M | Early childhood caries | Full mouth rehabilitation | GA | ASA I | Successful |
| P046 | 7-10 | F | Extensive decay | Extractions + space maintainer | GA | ASA II | Successful |
| P047 | 7-10 | F | Failed behavioral management | Multiple SSC + restorations | GA | ASA I | Successful |
| P048 | 7-10 | M | Special healthcare needs | Pulpectomy + restorations | Deep Sedation | ASA I | Successful |
| P049 | 7-10 | F | Acute dental infection | Incision & drainage + extractions | GA | ASA II | Minor complication - resolved |
| P050 | 7-10 | F | Multiple teeth requiring extraction | Restorations only | GA | ASA I | Successful |

***Notes:*** This table presents a representative sample of 50 cases from the total study population (n=385). All identifying information has been removed to ensure patient confidentiality.

**Abbreviations:** ECC = Early Childhood Caries; SSC = Stainless Steel Crown; GA = General Anesthesia; ASA = American Society of Anesthesiologists Physical Status Classification.

**Age Groups:** <3 years (n=90, 23.4%); 3–6 years (n=171, 44.4%); 7–10 years (n=124, 32.2%).

**Inclusion Criteria:** Pediatric patients (aged 0–10 years) referred for dental treatment under general anesthesia or deep sedation due to: (1) extensive dental treatment needs, (2) inability to cooperate with conventional dental treatment, (3) special healthcare needs, or (4) failed previous behavioral management attempts.

**Exclusion Criteria:** Patients with incomplete medical records, those who declined participation, and cases where the parent/guardian did not complete the questionnaire.

**Ethical Considerations:** All data were collected in accordance with the Declaration of Helsinki. Informed consent was obtained from all parents/guardians. The study protocol was approved by the Institutional Ethics Committee.

**Supplementary Table S2. Summary of Clinical Characteristics (n=385)**

| **Characteristic** | **n** | **%** |
| --- | --- | --- |
| **Primary Diagnosis** |  |  |
| Severe Early Childhood Caries (S-ECC) | 156 | 40.5 |
| Multiple carious lesions | 98 | 25.5 |
| Dental trauma requiring intervention | 42 | 10.9 |
| Failed behavioral management | 51 | 13.2 |
| Special healthcare needs | 38 | 9.9 |
| **Procedures Performed** |  |  |
| Restorations (composite/amalgam/GIC) | 312 | 81.0 |
| Extractions | 245 | 63.6 |
| Pulp therapy (pulpotomy/pulpectomy) | 178 | 46.2 |
| Stainless steel crowns | 134 | 34.8 |
| Space maintainers | 67 | 17.4 |
| **Anesthesia Type** |  |  |
| General Anesthesia | 298 | 77.4 |
| Deep Sedation | 87 | 22.6 |
| **ASA Physical Status Classification** |  |  |
| ASA I (healthy patient) | 289 | 75.1 |
| ASA II (mild systemic disease) | 96 | 24.9 |
| **Treatment Outcome** |  |  |
| Successful (no complications) | 371 | 96.4 |
| Minor complications (resolved) | 14 | 3.6 |
| Major complications | 0 | 0.0 |

***Notes:*** Procedure categories are not mutually exclusive; patients may have received multiple procedures in a single session. Minor complications included transient postoperative nausea/vomiting (n=8, 2.1%), prolonged bleeding requiring additional hemostatic measures (n=4, 1.0%), and mild soft tissue injury (n=2, 0.5%). All complications were managed successfully with no long-term sequelae. GIC = Glass Ionomer Cement; ASA = American Society of Anesthesiologists.
