# Supplementary material for: The role of social media in parents’ approaches to dental treatment procedures under general anesthesia and sedation: a cross-sectional survey in Turkey
Source: BMC Oral Health. 2026 Feb 2;26:409. doi: 10.1186/s12903-026-07779-9 (PMC12952005; doi:10.1186/s12903-026-07779-9)
Supplement: Supplementary file 3 — Supplementary Material 3. [file 12903_2026_7779_MOESM3_ESM.pdf]

Editör Comments

1] Please provide same data availability statement on system as provided in main manuscript.

2]We noticed that you have provided patience gender , age group and diagnosis which consider as personal information for that reason please provide consent for publication.

3] Please note that in the case of minors, which refers to individuals younger than the age of 16, consent to participate must be obtained from their parents or legal guardians. As such, we ask that in your 'Ethics approval and consent to participate' section, to clarify whether informed consent to participate was obtained from the parents or legal guardians of any participant under the age of 16 and clearly state this in your manuscript.

4] Please note that in the case of minors, which refers to individuals younger than the age of 18, written consent for publication must be obtained from their parents or legal guardians. As such, we ask that in your "Consent for publication" section, to please clarify whether written informed consent for publication of clinical details and/or clinical images was obtained from the parents of the patient.

5]At this stage, please upload your manuscript as a single, final, clean version that does not contain any tracked changes, comments, highlights, strikethroughs or text in different colours. All relevant tables/figures/additional files should also be clean versions and should remain uploaded as separate files. Please ensure that all figures, tables and additional files are cited appropriately within the text.

Response to Editör

1. Thank you for your comment. As requested, the same data availability statement provided in the main manuscript has now been added to the submission system.

2. Thank you for your comment. We hereby confirm that we give our consent for publication of the anonymized clinical data presented in this manuscript.

3. Thank you for your clarification. In the "Ethics approval and consent to participate" section, it is now clearly stated that informed consent to participate was obtained from the parents or legal guardians of participants under the age of 16.

4. Thank you for your comment. In the "Ethics approval and consent to participate" section, We clarify that written informed consent for publication of clinical details and/or clinical images was obtained from the parents of patients under the age of 18.

5. Thank you for your instruction. All required revisions have been completed and the manuscript has now been uploaded as a single, final, clean version without tracked changes, comments, highlights, strikethroughs, or colored text. All relevant tables, figures, and supplementary files have also been uploaded as clean versions in separate files, and they are appropriately cited within the text.
